# Acute and chronic functional and traditional resistance training improve muscular fitness in young males via the AMPK/PGC-1α/irisin signaling pathway

**DOI:** 10.1265/ehpm.23-00146

**Published:** 2023-11-15

**Authors:** Chongwen Zuo, Xiaoyan Ma, Chaoqun Ye, Zhiyang Zheng, Shumin Bo

**Affiliations:** 1 Air Force Medical Center of Chinese PLA, Beijing, 100142, China; 2 Capital University of Physical Education and Sports, Beijing, 100191, China; 3 Tianjin University, Tianjin, 300072, China; 4 Beijing Sports University, Beijing, 100091, China

**Keywords:** Resistance training, Muscular fitness, AMPK, PGC-1α, Irisin

## Abstract

**Background:**

In this study, we aimed to investigate the effects of acute and chronic resistance training of varying intensities on molecular responses and their association with muscular fitness in a cohort of young males who participated in this intervention study.

**Methods:**

Young males (19–28 years) with no prior training experience underwent a six-week program consisting of two distinct modalities of resistance training. The participants were randomly divided into a functional resistance training group (FRT; n = 9; participants performed 4–5 sets of 20 repetitions maximum (RM) at 40% 1RM) or a traditional resistance training group (TRT; n = 9; participants performed 4–5 sets of 12 RM at 70% 1RM). Both protocols entailed training three days per week for six weeks. Blood samples were obtained before, immediately after an acute bout of training, and after the six-week training program to determine alterations in molecular responses. Muscular fitness analysis and anthropometric measurements were conducted before and after the six-week training program.

**Results:**

After the six-week training program, the lean body mass of participants in both TRT and FRT groups was significantly increased (*p* < 0.05), whereas body fat percentage and fat mass were significantly decreased solely in the FRT group (*p* < 0.05). All muscular fitness variables were significantly increased in both groups (*p* < 0.01), with no difference between the two groups. Additionally, in the TRT group, serum levels of AMP-activated protein kinase (AMPK) were significantly increased following acute training and six weeks of resistance training, whereas in the FRT group, no significant increase in serum levels of AMPK was observed. In both groups, serum levels of peroxisome proliferator-activated receptor-γ coactivator-1α (PGC-1α), irisin, and insulin-like growth factor-1 were significantly increased. Moreover, myostatin was significantly decreased following acute training and six weeks of resistance training (*p* < 0.05), with no difference between the two groups. Furthermore, a significant correlation was observed between barbell back squat and certain molecular variables.

**Conclusions:**

Overall, our study indicates that acute and chronic resistance training of varying intensities are effective changing molecular responses, the chronic FRT and TRT improve muscular fitness in young males through the AMPK/PGC-1α/irisin signaling pathway.

**Trial registration:**

Chinese Clinical Trial Registry: ChiCTR2200059775 (11/05/2022).

## Introduction

According to the National Strength and Conditioning Association, the American College of Sports Medicine (ACSM), and the World Health Organization, limiting sedentary behavior and regularly engaging in resistance exercise training is integral to improving muscle mass and strength in adults [1–3]. However, the cellular and molecular mechanisms at play are yet to be comprehensively understood. Mitochondrial biogenesis and energy metabolism in skeletal muscles induced by physical exercise mechanically contribute to improved overall health [4]. These cellular responses and their adaptive alterations are substantially influenced by exercise protocols, such as exercise models, intensity, duration, and frequency [5]. Functional resistance training (FRT), which is a form of exercise that involves low-to-moderate loads while performing exercises on unstable surfaces, requires high levels of motor control and the production of muscle force necessary to overcome the external loads while maintaining stability [6]. FRT may be as effective as traditional resistance training (TRT) for improving movement ability, central body strength, and neuromuscular efficiency [7]. In humans, resistance training causes adaptive changes in the mitochondria, such as increased mitochondrial biogenesis and energy metabolism, which are closely associated with training intensity [8]. Therefore, a signal transduction pathway associated with mitochondrial biosynthesis may mediate the mechanism through which exercise improves muscle function.

During exercise, several signaling pathways are activated in the skeletal muscle. Notably, in vertebrates, the primary signaling pathway that stimulates mitochondrial biogenesis and oxidative metabolism is the AMP-activated protein kinase (AMPK)–peroxisome proliferator-activated receptor-γ coactivator-1α (PGC-1α) pathway [9, 10]. AMPK serves as a receptor of intracellular energy change, i.e., it is an intracellular energy sensor that is typically activated by cellular stressors, such as a decrease in ATP concentration and the subsequent increase in the AMP/ATP ratio induced by exercise [11]. This kinase is an upstream regulator of PGC-1α and plays a crucial role in the maintenance of intracellular energy homeostasis [12]. PGC-1α, a transcriptional coactivator closely associated with the regulation of the body’s energy metabolism, is primarily expressed in tissues that exhibit high energy requirements or abundant mitochondria, such as those of the heart, skeletal muscle, liver, and brown fat. It is closely associated with exercise-induced adaptive alterations such as mitochondrial biogenesis, muscle fiber type transformation, energy metabolism, and angiogenesis. Studies involving human muscle biopsies obtained from untrained healthy males have shown that a single bout of high-intensity exercise upregulates AMPK expression [13]. AMPK activates PGC-1α, thereby serving as a crucial regulatory factor of PGC-1α, and enhances mitochondrial biosynthesis in skeletal muscle and fatty acid oxidation [14]. Therefore, AMPK is currently known as the primary mediator of the exercise-induced PGC-1α signaling cascade. In addition, PGC-1α has been reported to regulate certain myokines; irisin is a recently identified exercise-induced and PGC-1α dependent myokine, discovered by Bostrom et al. [15]. They revealed that PGC-1α induces the expression of fibronectin domain-containing protein 5 (FNDC5), a membrane protein that undergoes cleavage and subsequent liberation from muscle tissue in the form of the hormone irisin [15]. Irisin serves as a messenger molecule between skeletal and peripheral tissues or organs, thereby leading to energy consumption, browning of subcutaneous white adipocytes, and the regulation of glucose and lipid metabolism via uncoupling protein 1, a thermogenic factor involved in the browning of white and beige adipocytes [15–17]. An increase in irisin concentration promotes oxygen consumption, improves glucose tolerance and insulin sensitivity, and facilitates weight loss in individuals engaged in training compared to those who do not engage in training [15]. However, despite recent studies, there are still controversies surrounding the precise role of irisin in exercise-related physiological changes in humans [18]. Therefore, further investigation is necessary to understand the function of irisin in metabolic regulation, body composition, and muscle function.

At present, resistance training is known to yield benefits comparable to endurance training in terms of enhancing the body’s energy metabolism and physical fitness; however, there is a lack of research on whether resistance training is equally effective in activating the AMPK/PGC-1α/irisin pathway. Studies have shown that resistance training induces distinct adaptations compared to endurance training, including muscle hypertrophy associated with increased muscle strength and power [19, 20]. Although earlier studies have elucidated that resistance training has a minimal impact on oxidative metabolism, more recent studies have shown that resistance training improves mitochondrial function in skeletal muscles [21, 22]. In addition, there is evidence indicating that acute resistance training increases PGC-1α expression and circulating irisin concentration [23]. Likewise, Liu et al. [24] validated that strength training prevents skeletal muscle atrophy and improves motor function through the PGC-1α/irisin pathway in aged rats. This suggests that adaptive regulation of skeletal muscle function may be due to the upregulation of PGC-1α and irisin expression as a result of muscle exercise [25]. Through in vitro experiments, Huh et al. [26] showed that irisin-stimulated myocytes exhibit insulin-like growth factor-1 (IGF-1) expression and inhibited myostatin (MSTN) expression, thereby contributing to muscle growth and potentially improving muscle function. Above all, these findings suggest that the underlying mechanism of irisin may potentially be a promising avenue for developing therapeutic strategies for metabolic disorders and enhancing skeletal muscle function [27].

To the best of our knowledge, a growing body of evidence suggests that exercise is the best approach to prevent a decline in muscle function across demographic groups and improve or sustain overall health [28]. In particular, resistance training has been validated to be an optimal method for enhancing muscle function [29] and body composition [30]. However, different models of resistance training to improve human health are lacking, and the cellular and molecular mechanisms underlying resistance training are yet to be fully elucidated [31]. In this study, to determine whether resistance training affects the AMPK/PGC-1α/irisin pathway and whether this signaling pathway is associated with muscular fitness, we compared the effects of acute and chronic FRT and TRT of varying intensities on the serum levels of AMPK, PGC-1α, irisin, IGF-1, and MSTN, as well as body composition and muscular fitness. Furthermore, we also aimed to investigate the correlation between molecular responses and muscular fitness variables. We hypothesized that compared to TRT, FRT would lead to a greater increase in the serum levels of AMPK, PGC-1α, and irisin. Additionally, we postulated that the AMPK/PGC-1α/irisin pathway would exhibit a correlation with muscular fitness.

## Methods

### Study design

This study employed a randomized controlled experimental methodology, incorporating both acute and chronic designs. All participants were recruited from the Capital University of Physical Education and Sports. The study was conducted in accordance with the university’s Institutional Review Board guidelines (Chictr.org ID: ChiCTR22000597750). The study spanned from May 2022 to July 2022. The primary researcher of this study ensured that all participants were adequately informed about the study procedures and the safety of the experiments and training protocols. Written informed consent was obtained from all participants. Furthermore, the participants were required to fill out a comprehensive medical history questionnaire, which was approved by the Ethics Committee of the university and conducted in accordance with the ethical guidelines outlined in the Declaration of Helsinki.

### Participants

A total of 20 male university students (19–28 years old) who were not majoring in sports were recruited through printed advertisements and word-of-mouth. The participants had an inactive lifestyle and had basic training experience but lacked systematic resistance training experience. Participants were informed of the purpose of the study and the potential risks before written informed consent for participation was obtained from them. Participants were selected for this study based on the following criteria: (1) non-sports major males (18- to 30-year-old) with an inactive lifestyle; (2) individuals without upper- or lower-body injury or orthopedic issues, as determined by a physician; (3) individuals not having participated in resistance training or any other exercise programs in the six months prior to the study; (4) individuals without cancer, hepatic disorders, renal disorders, osteoarthritis, or chronic diseases (diabetes, hypertension, or musculoskeletal dysfunction). Exclusion criteria were as follows: (1) individuals with a habit of smoking and alcohol consumption; (2) individuals with overt chronic diseases, inflammatory conditions, and contraindications to exercise; (3) individuals who consumed any supplements or medications in the six months prior to the study.

### Procedure

Participants were randomly assigned to either the TRT group or the FRT group and underwent one bout of acute training and six consecutive weeks (three days per week) of training. Before randomization, a statistician employed a simple randomization method to generate random numbers using Excel software and blinded data. Then, the participants were randomly divided into the FRT and TRT groups, with all participants agreeing to the group allocation. Participants visited the laboratory on three separate occasions, during which we obtained fasting venous blood in pyrogen-free tubes for subsequent molecular analyses. One-repetition maximal strength (1RM) assessment was performed prior to the first visit to determine the training intensity. During the first visit (pre-test), which was scheduled in the morning, all participants were required to provide fasting venous blood samples before engaging in resistance training (detailed below). Then, the participants visited the laboratory immediately upon completing the first training session (acute post-test) to provide blood samples (the second visit). The resistance training program spanned six consecutive weeks. Subsequently, 48 h after the final training session (chronic post-test), participants provided blood samples in the morning following an overnight fasting period and abstinence from caffeinated drinks and alcohol samples (the third visit). Additional assessments (including those of 1RM, muscular endurance, power, and body composition) were performed at baseline and after the six-week training period to determine the effects of the resistance training programs on anthropometric measurements and muscular fitness, as described in our previous study [32]. Additionally, all participants were instructed not to consume supplements or medications during the study period. Notably, none of the participants underwent a systematic training program in the year leading up to the study. However, it is important to acknowledge that we could not prohibit the participants from engaging in additional sports activities during the formal semester as they were students of a sports university.

**Fig. 1:**
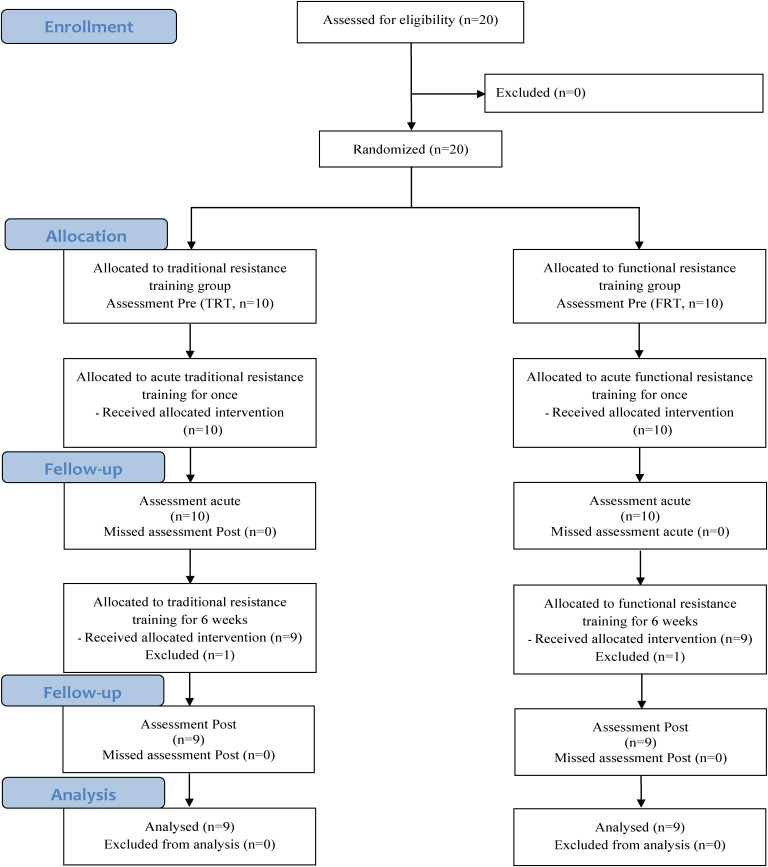
Flow chart illustrating the design of the study

Of the 20 participants, 1 participant in the TRT group withdrew from the study owing to personal reasons, while another participant in the FRT group also withdraw from the study citing personal reasons. Therefore, the study sample consisted of a total of 18 participants, and the experimental design is illustrated in Fig. 1. The characteristics of the participants are provided in Table 1.

**Table 1:** Baseline characteristics of the participants.

**Variables**	**Total (n = 18)**	**TRT (n = 9)**	**FRT (n = 9)**
Age (years)	23.7 ± 1.9	23.2 ± 2.0	24.2 ± 1.8
Height (cm)	176.8 ± 4.2	176.4 ± 4.4	177.2 ± 4.2
Body weight (kg)	75.4 ± 6.6	74.3 ± 7.2	76.5 ± 6.3
BMI (kg/m^2^)	24.1 ± 1.9	23.9 ± 2.4	24.3 ± 1.2

BMI, body mass index; TRT, traditional resistance training; FRT, functional resistance training. Data are presented as mean ± SD.

### FRT and TRT programs

A comprehensive illustration of the two types of training programs can be found in the article published by Zuo et al. [32]. In brief, each training session was supervised by an experienced trainer, spanned six consecutive weeks, and consisted of a whole-body workout conducted three times per week, with a minimum of 48 h between sessions. In each training session, the participants in the TRT group performed 4–5 sets of 12 repetitions at 70% of their 1RM of barbell squats, bench presses, deadlifts, leg curls, and reverse arm curls, with at least 1–2 min of rest between sets. Participants in the FRT group performed similar exercises as those performed in the TRT group, but on an unstable surface. The bench press exercise was performed in a supine position on a Swiss ball, and barbell back squats and deadlifts were performed while standing on a BOSU ball. Bulgarian split squats and kettlebell swings were performed while standing on a balance disc. Participants in the FRT group performed 4–5 sets of 20 repetitions at 40% 1RM of the corresponding exercises, with the same rest period between each set. Additionally, to ensure that participants readjusted their training intensity in response to their enhanced strength, we re-evaluated 1RM after three weeks of training. Our previous findings indicate that six weeks of FRT and TRT involving these exercises result in slightly greater muscular fitness and an improvement in body composition [32].

### Collection of blood samples

Blood samples were obtained from the participants at three different time points: before the commencement of the first training session (pretest), immediately after the first training session (acute post-test), and 48 h after the final training session (chronic post-test). Blood (5 mL) was drawn from a cubital vein into pyrogen-free tubes without EDTA by venipuncture. Notably, sampling was performed after approximately 12 h of fasting. Blood samples were then centrifuged at 3000 rpm for 15 min at 4 °C, and serum was transferred to a 200-µL microcentrifuge tube and stored at −80 °C until further analysis. Serum levels of AMPK, PGC-1α, irisin, IGF-1, and MSTN were analyzed using enzyme-linked immunosorbent assay kits for humans (Shanghai Meilian Bioengineering Institute, Shanghai, China), with a sensitivity of 1.0 ng/mL, 1.0 nmol/L, 1.0 ng/mL, 1.0 ng/mL, and 0.1 ng/mL, respectively. The intra- and inter-assay coefficients of variability for all blood markers were less than 10% and 15%, respectively.

### Anthropometric measurements

The participants’ body weight and height were measured using a wall-mounted stadiometer and a medical scale, respectively. The measurements were performed with the participants wearing lightweight clothing and no footwear. The body composition, including body weight, body fat percentage, fat mass, and lean body mass, was assessed before and after the six-week training period. This was accomplished using a bioelectrical impedance device (Tanita MC-980MA, Tokyo, Japan), which has been validated to measure alterations in human body composition with a high degree of accuracy [33]. Body mass index (BMI) as calculated using the following equation: BMI = weight (kg)/height (m)^2^. All anthropometric assessments were evaluated by a researcher who was experienced and qualified to perform the assessments. The participants were instructed to fast, abstain from consuming any food or beverages, and refrain from physical activity in the 24-h period leading to bioelectrical impedance analysis.

### One-repetition maximal strength assessment

Before commencing the training program, each participant underwent a one-repetition maximal strength test for each exercise as previously described [34]. One week before the acute bout of training, maximal muscular strength tests were conducted to determine the 1RM of four exercises (barbell back squat, bench press, deadlift, and leg flexion). Each test was performed according to the guidelines prescribed in the ACSM [35]; participants lifted progressively increasing weight until failure. Briefly, the participants performed two sets of 8–10 repetitions at 40–60% 1RM as a warm-up, followed by a second round of warm-up, which consisted of one set of 3–5 repetitions at approximately 60–80% 1RM, and a third round of warm-up, which included 1–3 repetitions at 90% 1RM. A resting period of 1–2 min was permitted between warm-up sets. Following the warm-up, each participant underwent the 1RM test. Five trials were conducted, whereby the load was progressively increased in each consecutive trial until the participants were unable to perform a proper lift. A rest period of 3 min was allowed between each trial. The highest load lifted in the final attempt was recorded as the 1RM load.

Before the actual test, each participant was presented with a demonstration and instructed on how each exercise was to be performed. The bench press exercise is a valid method for assessing the strength of the upper limbs. This exercise required the participants to first fully extend their arms and grasp the bar directly above their chest. Subsequently, they had to gradually lower the bar at a controlled speed and in a smooth manner towards their chest without contacting their chest, and then return the bar to the starting position. The barbell back squat is used as a valid method to assess lower-limb strength. Initially, the participants held a bar on their back while in a standing position and their core fully stretched. Subsequently, they positioned their core perpendicular to the knee with their feet shoulder-width apart at a 45° angle and their knees at a 90° angle. Then, they immediately straightened their legs. A deadlift was performed by lifting a barbell. The participants stood close to the middle of the bar with their feet shoulder-width apart. Subsequently, they squatted down to grasp the bar with an alternative grip, their arms positioned straight and slightly wider than the width of their shoulders, and their hips positioned lower than their shoulders. Starting in the squat position, they lifted the target weight placed on the ground by extending their hips and knees while keeping their backs straight until they achieved a fully upright position. The leg flexion test was selected to gather data on maximal dynamic strength exhibited throughout the full range of motion of the involved muscles. A leg flexion test was completed using a standard leg flexion machine with the participants in a seated position (the hip angle was approximately 110°). Under the guidance of the researchers, participants performed a concentric flexion with their dominant leg, starting from an extended position of 180° and concluding at an approximate flexion of 70°.

### Power-countermovement jump (CMJ) assessment

Lower-limb power performance was assessed using the CMJ test (Quattro Jump System, Kistler, Switzerland). Participants performed CMJs while refraining from positioning their arms in an akimbo stance on a portable force plate. This test commenced with the participant assuming an upright stance with their arms at the sides of their body on the force plate. Upon receiving the verbal cue “start” from the instructor, the participants rapidly flexed their knees until reaching a 90° angle and then vertically jumped as high as possible while swinging their arms, as described previously [36]. Each participant performed three consecutive trials with a 30-s rest period in between the trials, and the highest jump among the three jumps was selected for further analysis.

### Muscular endurance assessment

To assess muscular endurance, we instructed participants to perform bench press and leg flexion exercises, aiming to complete as many repetitions as feasible at 70% 1RM until they reached the point of volitional failure, similar to that described in our previous study [7]. Before and after the six-week training period, muscular endurance (70% of their baseline 1RM) was measured; the 1RM test for each exercise was followed by a 10-min resting period. Throughout the testing process, the repetition cadence was approximately 1.5 s for concentric and eccentric contractions and was controlled by an electronic metronome. The test was concluded when participants were unable to keep up with the metronome’s pace. Furthermore, the range of motion and techniques for bench press and leg flexion exercises were similar to those described above. The maximum number of repetitions and volume load were recorded for further analysis.

### Statistical analysis

All statistical analyses were performed using GraphPad Prism software (version 8.4; GraphPad, San Diego, CA, USA). All data are presented as mean ± standard deviation (SD) and validated to be normally distributed using both the Kolmogorov–Smirnov test and Shapiro–Wilk’s W tests before being subjected to subsequent statistical analyses. Independent-samples *t*-test was employed to examine the inter-group differences in baseline data. A two–way repeated measure analysis of variance (time × group) was employed to analyze the differences in anthropometric measurements, muscular fitness, and molecule index. In cases the interaction effect was significant, simple main effect analysis was performed using post hoc tests. Furthermore, Cohen’s d effect sizes were calculated as partial eta square (η^2^), as proposed by Cohen [37], and categorized as small (<0.01), moderate (0.01–0.138), or large (>0.138) for all data. In addition, the Pearson correlation coefficient was used for evaluating the correlations between molecular responses and muscular fitness variables changes (differences in delta values between posttests and pretests). *p* < 0.05 was considered to indicate statistical significance.

## Results

### Characteristics and body composition of the participants

The number of participants recruited in this study is shown in Fig. 1. Of the 20 participants who joined the study, 2 withdrew from the study owing to personal reasons after group allocation and the introduction of the training programs. Therefore, 18 participants were included in the final analysis. Participants in both the FRT and TRT groups completed 100% of the training sessions. No significant differences in body composition were observed between the two groups before the training program. Muscular fitness analysis showed no significant differences in 1RM, muscular endurance, or power (all *p* > 0.05) before the training program between the two groups. Additionally, no training-related injuries occurred in either the TRT or FRT groups.

Table 2 shows the mean values of body composition parameters before and after the six-week training program for both the FRT and TRT groups. No differences in body weight, BMI, percentage of body fat, fat mass, or lean body mass were observed between the FRT and TRT groups before the training intervention (*p* > 0.05). After the six-week training program, the percentage of body fat and fat mass was significantly decreased compared with baseline (*p* < 0.05) in the FRT group, and both groups exhibited a significant increase in lean body mass (*p* < 0.05) but no significant difference was observed between the two groups. Additionally, no significant effects of time × group interactions were observed on weight (*p* = 0.776, η^2^ = 0.005), BMI (*p* = 0.083, η^2^ = 0.177), lean body mass (*p* = 0.740, η^2^ = 0.014), percentage of body fat (*p* = 0.345, η^2^ = 0.056), and fat mass (*p* = 0.535, η^2^ = 0.024).

**Table 2:** Alterations in body composition following six weeks of resistance training

**Variable**		**Pre**	**Post**	**Two-way ANOVA**		
				Interaction	*p*-value	η^2^

Weight (kg)	TRT	74.3 ± 7.1	73.5 ± 6.0	Time	0.146	0.127
	FRT	76.5 ± 6.3	76.0 ± 5.5	Group	0.432	0.039
				Time × group	0.776	0.005
BMI (kg/m^2^)	TRT	23.9 ± 2.4	23.2 ± 2.4*	Time	0.000	0.550
	FRT	24.3 ± 1.2	24.0 ± 1.2	Group	0.479	0.032
				Time × group	0.081	0.178
Lean body mass (kg)	TRT	58.3 ± 4.0	59.1 ± 3.9*	Time	0.000	0.626
	FRT	61.4 ± 5.4	62.3 ± 5.4*	Group	0.173	0.113
				Time × group	0.640	0.014
Body fat (%)	TRT	16.4 ± 6.2	15.9 ± 5.6	Time	0.031	0.259
	FRT	16.8 ± 5.4	15.7 ± 5.1*	Group	0.986	0.000
				Time × group	0.345	0.056
Fat mass (kg)	TRT	12.5 ± 5.8	11.9 ± 4.9	Time	0.017	0.305
	FRT	13.0 ± 5.1	12.0 ± 4.7*	Group	0.745	0.007
				Time × group	0.535	0.024

ANOVA, analysis of variance; BMI, body mass index; TRT, traditional resistance training; FRT, functional resistance training; Pre, before intervention; Post, after intervention. * *p* < 0.05, Pre vs. Post. Data are represented as means ± SD.

### One-repetition maximal strength

The one-repetition maximal muscle strength of the participants is shown in Table 3. At baseline, no significant differences in muscle strength were observed between the FRT and TRT groups. After the six-week training program, the maximal strength of the upper and lower limbs was significantly increased compared with baseline in both the TRT and FRT groups (*p* < 0.01), with no significant difference between the groups. In addition, no significant effects of time × group interactions were observed for the bench press (*p* = 0.609, η^2^ = 0.017), barbell back squat (*p* = 0.707, η^2^ = 0.009), deadlift (*p* = 0.908, η^2^ = 0.001), and leg flexion exercises (*p* = 0.358, η^2^ = 0.053).

**Table 3:** Alterations in maximal muscle strength following six weeks of resistance training

**Variable**		**Pre**	**Post**	**Two-way ANOVA**		
				Interaction	*p*-value	η^2^

BP (kg)	TRT	79.7 ± 17.4	88.2 ± 17.9**	Time	0.000	0.895
	FRT	76.7 ± 13.5	84.4 ± 12.0**	Group	0.643	0.014
				Time × group	0.609	0.017
BBS (kg)	TRT	116.7 ± 17.4	134.0 ± 20.8**	Time	0.000	0.943
	FRT	120.3 ± 13.5	138.4 ± 15.1**	Group	0.644	0.014
				Time × group	0.707	0.009
DL (kg)	TRT	113.3 ± 20.6	127.8 ± 18.7**	Time	0.000	0.831
	FRT	114.7 ± 15.4	129.4 ± 13.1**	Group	0.843	0.003
				Time × group	0.908	0.001
LF (kg)	TRT	42.9 ± 6.4	47.9 ± 6.7**	Time	0.000	0.895
	FRT	39.4 ± 6.8	45.4 ± 5.5**	Group	0.327	0.060
				Time × group	0.358	0.053

ANOVA, analysis of variance; BP, bench press; BBS, barbell back squat; DL, deadlift; LF, leg flexion; TRT, traditional resistance training; FRT, functional resistance training; Pre, before intervention; Post, after intervention. * *p* < 0.05, ** *p* < 0.01, Pre vs. Post. Data are represented as means ± SD.

### Power-CMJ

The power output in CMJs performed by the participants is shown in Table 4. The CMJ was significantly increased following the six-week training program compared with the baseline in both the TRT and FRT groups (*p* < 0.05), but no significant differences were observed between the groups. Moreover, no significant effect of time × group interaction was observed for CMJ (*p* = 0.410, η^2^ = 0.043).

**Table 4:** Alterations in CMJ following six weeks of resistance training

**Variable**		**Pre**	**Post**	**Two-way ANOVA**		
				Interaction	*p*-value	η^2^

CMJ (cm)	TRT	61.2 ± 7.6	65.1 ± 6.4*	Time	0.000	0.648
	FRT	66.8 ± 8.3	72.0 ± 7.7**	Group	0.089	0.170
				Time × group	0.410	0.043

ANOVA, analysis of variance; CMJ, counter movement jump; TRT, traditional resistance training; FRT, functional resistance training; Pre, before intervention; Post, after intervention. **p < 0.01, Pre vs. Post. Data are represented as means ± SD.

### Muscular endurance

Regarding muscular endurance, the repetitions of bench press and leg flexion were significantly increased following the six-week resistance training program in both groups (*p* < 0.01), with no significant difference between the groups. Moreover, muscular endurance, expressed as volume–load for bench press and leg flexion, was also significantly increased (*p* < 0.01), with no significant difference between the groups. Additionally, no significant time × group interactions were observed for the number of repetitions at 70% 1RM for the bench press (*p* = 0.138, η^2^ = 0.132) and the right leg flexion exercises (*p* = 0.096, η^2^ = 0.163) (Table 5).

**Table 5:** Alterations in BP and R-LF muscular endurance following six weeks of resistance training

**Variable**		**Pre**	**Post**	**Two-way ANOVA**		
				Interaction	*p*-value	η^2^

BP Rep	TRT	16.6 ± 3.0	21.9 ± 4.9**	Time	0.000	0.860
	FRT	14.3 ± 2.2	21.7 ± 3.1**	Group	0.423	0.041
				Time × group	0.138	0.132
BP VL (kg)	TRT	927.5 ± 276.8	1368.6 ± 342.7**	Time	0.000	0.889
	FRT	774.3 ± 193.9	1272.8 ± 280.1**	Group	0.332	0.059
				Time × group	0.500	0.029
LF Rep	TRT	21.0 ± 5.2	28.0 ± 6.2**	Time	0.000	0.848
	FRT	18.1 ± 4.9	28.3 ± 7.2**	Group	0.638	0.014
				Time × group	0.096	0.163
LF VL (kg)	TRT	623.6 ± 176.7	827.4 ± 202.6**	Time	0.000	0.864
	FRT	498.9 ± 162.0	777.7 ± 220.7**	Group	0.333	0.059
				Time × group	0.137	0.133

BP, bench press; LF, leg flexion; Rep, repetition; VL, volume-load; TRT, traditional resistance training; FRT, functional resistance training; Pre, before intervention; Post, after intervention. ** *p* < 0.01, Pre vs. Post. Data are represented as means ± SD.

### Biochemical characteristics

**Fig. 2:**
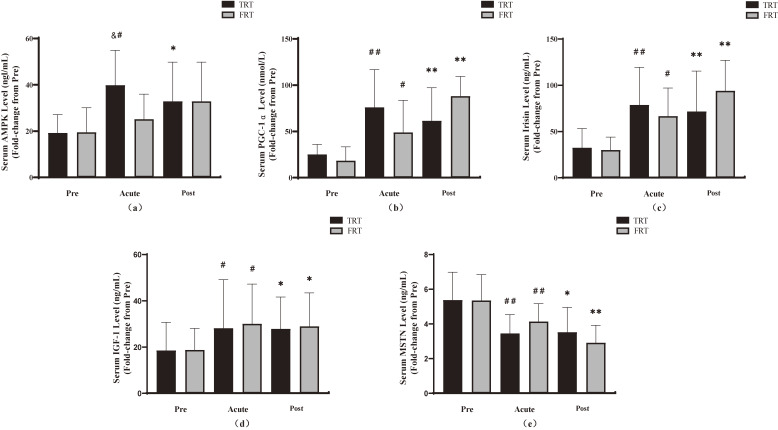
Effects of acute and chronic resistance training on the levels of blood markers. a, AMPK, AMP-activated protein kinase; b, PGC-1α, peroxisome proliferator-activated receptor-γ coactivator-1α; c, irisin; d, IGF-1, insulin-like growth factor-1; e, MSTN, myostatin. # p < 0.05, ## p < 0.01, Pre vs. Acute difference within group; & p < 0.05, Acute difference between groups; * p < 0.05, ** p < 0.01, Post vs. Pre difference within group. All data are represented as means ± SD. TRT, traditional resistance training; FRT, functional resistance training.

Prior to conducting the training program, no significant inter-group differences were observed in any of the measured biochemical values (*p* > 0.05); however, a time × group interaction (*p* < 0.05, η^2^ = 0.243) was observed for serum levels of AMPK. The post hoc tests for time main effect analyses showed that serum levels of AMPK in TRT group were significantly increased following acute and chronic resistance training (*p* < 0.05), whereas in the FRT group, no significant increase in serum levels of AMPK was observed (*p* > 0.05). In group comparison, serum levels of AMPK in TRT group were elevated to a greater degree than those in the FRT group following acute training (*p* < 0.05); however, no significant difference in serum levels of AMPK was observed between the groups following chronic training (Fig. 2a).

Time × group interaction (*p* < 0.05, η^2^ = 0.243) was observed for serum levels of PGC-1α. The post hoc tests for time main effect analyses showed that serum levels of PGC-1α were significantly increased following acute and chronic resistance training in the TRT and FRT groups (*p* < 0.05). In group comparison, no significant difference in serum levels of PGC-1α was observed between the two groups following acute and chronic resistance training (Fig. 2b).

The serum levels of irisin and IGF-1 were significantly increased following acute and chronic resistance training in both groups (*p* < 0.05) (Figs. 2c, 2d), and the serum levels of MSTN were significantly decreased compared with baseline values (*p* < 0.05) (Fig. 2e), but no significant difference was observed between the two groups. Furthermore, no significant time × group interactions were observed for serum levels of irisin (*p* = 0.170, η^2^ = 0.105), IGF-1 (*p* = 0.867, η^2^ = 0.005), and MSTN (*p* = 0.191, η^2^ = 0.100).

### Correlation between variables

**Fig. 3:**
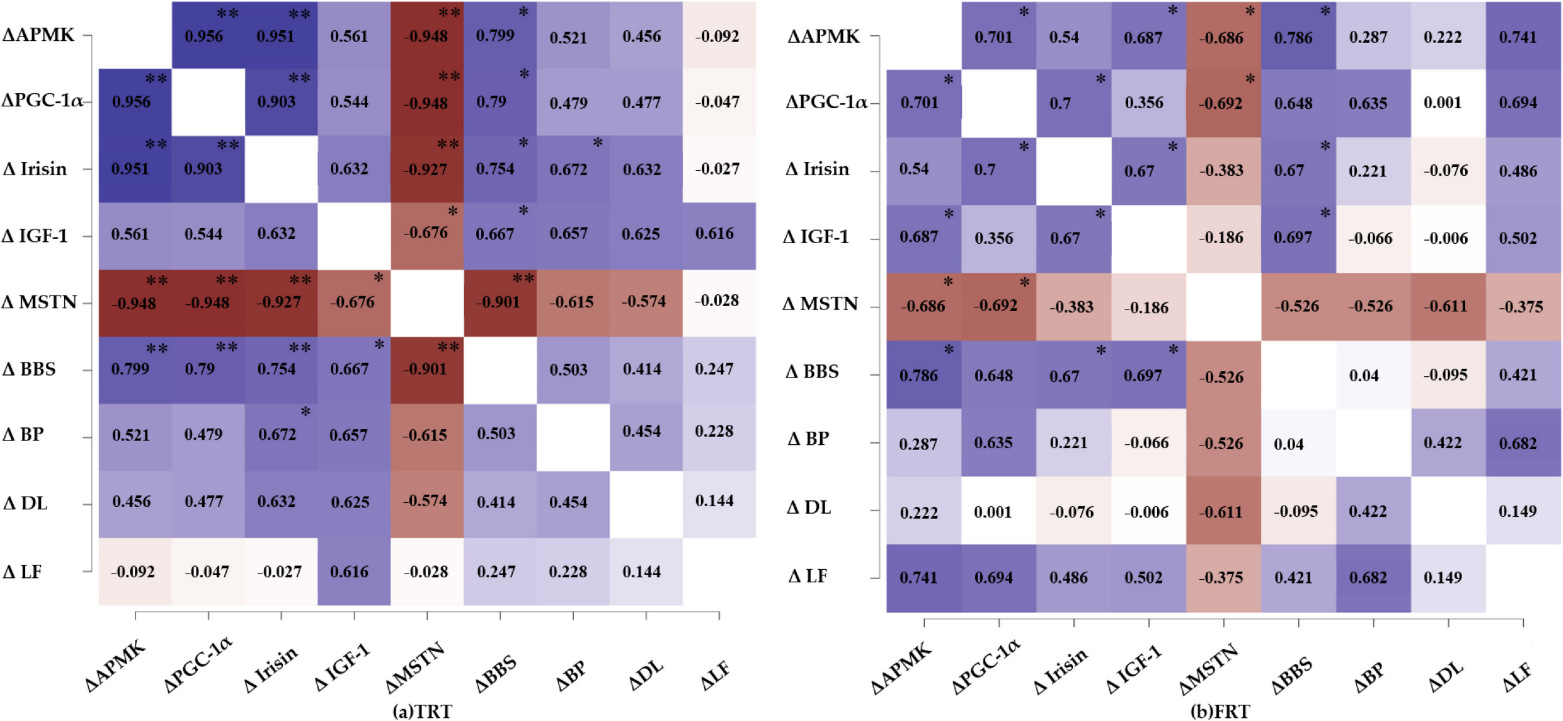
Pearson correlation coefficients heat mapping for delta value in maximal strength, serum AMPK, PGC-1α, irisin, IGF-1 and MSTN in TRT group (a) and FRT group (b). AMPK, AMP-activated protein kinase; PGC-1α, peroxisome proliferator-activated receptor-γ coactivator-1α; IGF-1, insulin-like growth factor-1; MSTN, myostatin; BBS, barbell back squat; BP, bench press; DL, deadlift; LF, leg flexion; significant correlation * p < 0.05, ** p < 0.01; TRT, traditional resistance training; FRT, functional resistance training.

Figure 3 shows the Pearson correlation coefficients (delta values) for maximal strength, the serum levels of AMPK, PGC-1α, irisin, IGF-1, and MSTN, and the associations among them. In both groups, based on the delta values, AMPK was positively correlated with PGC-1α (TRT group: *r* = 0.956, *p* < 0.01, Fig. 3a; FRT group: *r* = 0.701, *p* < 0.05, Fig. 3b); PGC-1α was positively correlated with irisin (TRT group: *r* = 0.903, *p* < 0.01, Fig. 3a; FRT group: *r* = 0.700, *p* < 0.05, Fig. 3b); and irisin was positively correlated with IGF-1 (TRT group: *r* = 0.632, *p* = 0.068, Fig. 3a; FRT group: *r* = 0.670, *p* < 0.05, Fig. 3b) and negatively correlated with MSTN (TRT group: *r* = −0.927, *p* < 0.01, Fig. 3a; FRT group: *r* = −0.383, *p* = 0.310, Fig. 3b). Furthermore, based on the delta values, a significant positive correlation was observed between the barbell back squat exercise and the serum levels of AMPK, PGC-1α, irisin, and IGF-1 (TRT group: *r* = 0.799, *p* < 0.01; *r* = 0.790, *p* < 0.05; *r* = 0.754, *p* < 0.05; *r* = 0.667, *p* < 0.05; respectively, Fig. 3a; FRT group: *r* = 0.786, *p* < 0.05; *r* = 0.648, *p* = 0.059; *r* = 0.670, *p* < 0.05; *r* = 0.697, *p* < 0.05; respectively, Fig. 3b) and negatively correlated with serum levels of MSTN (TRT group: *r* = −0.901, *p* < 0.01, Fig. 3a; FRT group: *r* = −0.526, *p* = 0.146, Fig. 3b). There is no significant correlation between bench press, deadlift, leg flexion and molecular variables.

## Discussion

Resistance training has been reported as the most effective approach to significantly improve muscle mass, size, and strength, as well as physical performance, in any type of individual [28, 38]. In this study, we compared the effects of two resistance training modalities on muscular fitness and the serum levels of proteins associated with mitochondrial biosynthesis in young males. Notably, this is the first evidence from human blood perspective proved that resistance training could improve muscular fitness via the serum AMPK/PGC-1α/irisin signal pathway. The major findings of this study are as follows: (1) FRT significantly reduces the percentage of fat and fat mass and increases lean body mass; (2) both FRT and TRT improve muscular fitness, including maximal strength, power, and muscular endurance; (3) both FRT and TRT result in significant short-term and long-term increases in the serum levels of AMPK, PGC-1α, irisin, and IGF-1 and significantly decrease the serum levels of MSTN; and (4) the delta changes of barbell back squat in both FRT and TRT are significantly correlated to certain molecular variables. These findings indicate that both TRT and FRT can facilitate muscular fitness by improving the body’s energy metabolism and promoting mitochondrial biogenesis.

Our previous study, which employed a randomized control trial identical to the one utilized in this study, indicated that resistance training increases muscular fitness and improves body composition on untrained young males. Although the body composition indices did not exhibit significant alterations, the results of the current study regarding the reduction in the percentage of body fat and the increase in lean body mass are similar to the results of our previous study [39]. In the previous study, we compared the effects of TRT and FRT in untrained young males. Both the resistance training protocols induced similar reductions in body fat percentage (−2.3% vs. −1.4% for TRT and FRT, respectively) and alterations in lean body mass (0.9 kg vs. 1.1 kg, respectively) [39], and similar results were also observed in this study, statistical analysis showed that both protocols reduced body fat percentage (−3.0% vs. −6.5% for TRT and FRT, respectively) and increased lean body mass (0.6 kg vs. 1.0 kg, respectively), suggesting that FRT seems to elicit greater potential physiological and metabolic responses [40]. As previously suggested that both acute and chronic exercise could significant up-regulate the PGC-1α, which created conditions for downstream irisin formation [41]. Irisin is known as a new exercise-induced myokines that plays an important mediating role in improving body composition [42]. It is also considered a signaling hormone for muscle derived energy expenditure, which could induce browning in adipose tissue, increase energy expenditure, promote fat burning, and thus improve body composition. Although the correlation between irisin and body fat percentage is not available in this study, our data suggest that it seems to be a link between them after resistance training, we believe that future study will further provide definitive answers.

Our study revealed no difference in muscular fitness following the training program between the two groups. Participants in both groups exhibited comparable upper and lower limb 1RM strength, muscular endurance, and power under similar training volume. These findings are consistent with our previous hypothesis that training under unstable conditions results in neuromuscular adaptations similar to those induced by stable conditions. Our data is not without support; Maté-Muñoz [43] found that resistance training under unstable conditions (using BOSU ball and TRX^®^) is as effective as TRT for enhancing maximal strength in bench press and back squat exercises in untrained young males. It is likely that skeletal muscle adaptations (strength and muscle mass) were influenced by resistance training of equal training volume but different protocols. On the other hand, in the FRT group, when forces were applied without overloading the upper and lower muscles under unstable conditions, the improvement in strength was probably associated with an increase in the activation of trunk and lower muscles [44], sympathetic transmission, and recruitment of motor neurons, thereby possibly endorsing intramuscular and intermuscular coordination and cooperation [45] and making agonist muscle activation more economic, ultimately enhancing strength. Additionally, another finding of this study is that FRT significantly increases an individual’s power and endurance. Several studies on resistance training suggest that the key determinant for improving muscle power, strength, and endurance, irrespective of the load applied, is the execution of exercises at high velocity with a high number of repetitions [35, 46]. The findings of this study emphasize that using instability devices, such as BOSU and Swiss balls, during high-repetition training increases power and muscle endurance. Therefore, FRT at low-to-moderate intensity with high repetitions improves power and muscle endurance, thereby serving as an effective training method.

AMPK, a highly conserved regulator of cellular energy metabolism, plays a crucial role in the regulation of cell growth and the maintenance of the body’s energy homeostasis. Since AMPK can be activated by alterations in the AMP/ATP ratio during exercise [47], it may increase mitochondrial biosynthesis and reinforce skeletal muscle [48]. This study revealed that serum levels of AMPK increase after acute and chronic TRT and FRT, although FRT does not significantly increase serum levels of AMPK. Recently, certain forms of aerobic exercise have been found to promote the expression of AMPK [49, 50]; however, studies exploring the effect of resistance training on AMPK are limited. The aforementioned findings of our study are almost consistent with those of a previous study, which demonstrated that AMPK is regulated by exercise intensity in a rat exercise model; specifically, the high-intensity group exhibited greater AMPK activity, whereas the AMPK activity remained unchanged in the low-intensity group, indicating that AMPK activity is positively correlated to exercise intensity [51]. Furthermore, Dreyer et al. [52] reported that AMPK increases by 75% immediately following a bout of resistance exercises, and Rasmussen and Winder [53] indicated that AMPK activity is closely associated with exercise intensity and that the extent of its activation depends on exercise load.

Nonetheless, the potential reasons for the lack of a significant increase in serum levels of AMPK following FRT may be as follows: first, FRT may not increase the AMP/ATP ratio; second, low-intensity FRT only engages a portion of the muscle fibers exhibiting high AMPK activity, whereas AMPK activity in other portions of the engaged muscle fibers remains unchanged, resulting in no significant change in the activity of the entire muscle [54]. Moreover, during exercises of moderate to high intensity, AMPK activation is also affected by various factors, such as the increase in the AMP/ATP ratio and creatine/phosphocreatine ratio, the reduction in muscle glycogen content, hypoxia, and the accumulation of acidic substances; among these factors, the AMP/ATP ratio and acidic substance accumulation are considered to be the most important [51]. In particular, TRT is characterized by high intensity, greater engagement of fast-twitch fibers, and a heightened energy metabolism rate compared to slow-twitch fibers; thus, TRT significantly alters the AMP/ATP ratio and increases lactate production, which is consistent with the notion that AMPK in fast-twitch muscle fibers is more readily activated than that in slow-twitch muscle fibers [55]. It also validates the notion that AMPK may be primarily regulated by alterations in the AMP/ATP ratio and the accumulation of acidic substances. The TRT protocol adopted in this study was possibly a sufficient stimulus to induce changes in the AMP/ATP ratio, as the intensity of the TRT protocol was higher than that of the FRT protocol, thereby resulting in a rapid increase in lactate concentration and a higher muscle glycogen consumption rate. Therefore, serum levels of AMPK after acute training were significantly higher after TRT than those after FRT, which is highly consistent with the alterations in AMPK activity. Owing to limited data, future studies should further determine the phase change in AMPK following acute and chronic resistance training of various modalities.

In this study, we investigated the effect of resistance training on serum levels of PGC-1α. The results indicated a significant increase in serum levels of PGC-1α, which is a key regulator of mitochondrial biogenesis, following six weeks of TRT and FRT. To the best of our knowledge, no study has compared the effects of various models or intensities of resistance training on serum levels of PGC-1α. Our findings are consistent with those of one study, which was conducted by Hooshmand-Moghadam et al. [56]; they reported that the serum levels of PGC-1α and telomerase enzyme increased following 12 weeks of resistance training at an intensity of 60% 1RM. Although there exists evidence indicating that the expression of PGC-1α is positively associated with aerobic exercise intensity [57], the mechanism underlying the increase in serum levels of PGC-1α in response to resistance training of varying intensity remains unclear. However, the increase in serum levels of PGC-1α may be due to the activation of AMPK. As mentioned above, the activation of AMPK depends on the alterations in the AMP/ATP ratio, training intensity, and acidic substance accumulation caused by exercise, which may increase the activity of AMPK. Moreover, accumulating evidence suggests that AMPK directly phosphorylates PGC-1α, which is an essential step for the liberation of PGC-1α from its receptors [48, 58]. In this study, serum levels of AMPK in both groups increased after acute and chronic training, indicating that the increase in PGC-1α expression is indeed induced by AMPK [59]. Moreover, the positive correlation between serum levels of AMPK and PGC-1α also validates these findings.

In this study, serum levels of irisin were positively correlated with those of PGC-1α in both groups, indicating that an increase in the serum levels of irisin is accompanied by an increase in PGC-1α expression following acute training and six weeks of training. This suggests that the regulation of irisin in the blood of humans involves its hydrolysis and cleavage from FNDC5 by proteolytic enzymes and its subsequent release into the bloodstream [60]. A study by Pekkala et al. [61] showed a surprising increase in irisin concentrations in the circulation but no significant change in muscle FNDC5 following acute resistance training and 21 weeks of combined aerobic and strength exercises, suggesting that compared to endurance training, resistance training affects the levels of irisin to a greater degree [23]. Additionally, Kim et al. [42] found that, in obese males, serum levels of irisin increase significantly following eight weeks of resistance training, whereas endurance training does not result in any changes. In contrast, a few studies have elucidated that long-term resistance training does not alter the levels of irisin. This apparent inconsistency in findings may be attributed to racial differences, exercise intensity employed in the studies, experimental design, method of detection sample size, gender, age, and physical condition [62]. Therefore, additional research is necessary to explore the effect of training of varying intensity or protocol on serum levels of irisin.

Skeletal muscle has been identified as an endocrine organ that regulates several physiological and metabolic pathways in response to muscle contraction. Certain myokines, such as IGF-1 and MSTN, may be involved in inducing skeletal muscle hypertrophy, enhancing strength, and suppressing skeletal muscle atrophy. Recent studies showed that an increase in circulating irisin is strongly correlated with an increase in IGF-1 levels and a decrease in MSTN levels [26, 63, 64]. An in vitro study also indicated that irisin treatment regulates the downstream molecules IGF-1 and MSTN [26]. IGF-1 and MSTN have been identified as positive and negative regulators of muscle growth, respectively [65], and their regulation by irisin implies that irisin may enhance muscle mass and strength. These findings are validated by our study, wherein both FRT and TRT groups exhibited significant improvement in muscular fitness following the six-week training program, and correlation analysis showed that irisin is not only positively correlated with lower extremity muscle strength but also positively or negatively correlated with IGF-1 or MSTN in both FRT and TRT groups. Additionally, analysis of the body composition showed that both FRT and TRT may contribute to lean body mass. This finding indicates a novel avenue for enhancing muscle strength through any type of resistance training regimen in males, which may be potentially applicable to other populations, such as individuals with muscle atrophy. To the best of our knowledge, to date, no study has elucidated the relationship between muscular fitness, molecule responses, and resistance training of varying intensities in untrained males with an inactive lifestyle during acute and chronic training. The relevance of specific mechanisms underlying the effects of these molecules requires further exploration.

Our study has several limitations. First, given to the small sample size and the involvement of only young males as participants might limit the applicability of our findings to a wider population. Future studies should delve into the effects of TRT and FRT on large sample, including female participants and individuals with sarcopenia. Additionally, the lack of daily food intake records of these participants is a shortcoming in this study. Therefore, this should be considered more concretely and carefully when interpreted the results. Secondly, owing to the participants’ concerns regarding muscle biopsy techniques, we only analyzed blood samples. Muscle biopsy techniques are essential for accurately measuring protein and mRNA levels of AMPK and PGC-1α through western blotting and polymerase chain reaction analysis, which could further support our results at molecular level. Furthermore, this is the first study to compare the influence of resistance training of varying intensities on the regulatory factors associated with mitochondrial synthesis in the skeletal muscle of humans. Although our study did not reveal any differences in the AMPK/PGC-1α/irisin pathway across resistance training protocols of varying intensities, it suggests that resistance training may mitigate muscle function decline by activating the AMPK/PGC-1α/irisin pathway.

## Conclusions

In conclusion, participation in different types of resistance training has a positive potential effect on the improvement of muscular fitness and prevention of frailty [66]. The acute and chronic FRT and TRT exercises increase the serum levels of AMPK, PGC-1α, irisin, IGF-1, and decrease MSTN in young males. Meanwhile, the chronic FRT and TRT improve muscular fitness, which may be mediated by the AMPK/PGC-1α/irisin pathway at the molecular level. Additionally, our study suggests that individuals who engage in FRT exhibit improvements in body composition similar to individuals engaged in TRT. We believe that the findings of this study provide novel insights and useful training method in young males for preventing functional decline and keeping health condition from social medicine and preventive medicine perspective.

## Abbreviations

FRT: Functional resistance training
TRT: Traditional resistance training
AMPK: AMP-activated protein kinase
PGC-1α: Peroxisome proliferator-activated receptor-γ coactivator-1α
1RM: One repetition maximum
FNDC5: Fibronectin domain-containing protein 5
IGF-1: Insulin-like growth factor-1
MSTN: Myostatin
BMI: Body mass index
CMJ: Counter movement jump
BP: Bench press
BBS: Barbell back squat
DL: Deadlift
LF: Leg flexion

## Declarations

### Availability of data and materials

All data generated and analyzed during this study are included in this published article. The raw data supporting the conclusions of this article will be made available by the authors, without undue reservation, to any qualified researcher. First author should be contacted if someone wants to request the data from this study.

## References

[1] Conley MS, Rozenek R. National Strength and Conditioning Association Position Statement. J Strength Cond. 2001;23:9.

[2] Jakicic JM, Clark K, Coleman E, Donnelly JE, Foreyt J, Melanson E, . American College of Sports Medicine position stand. Appropriate intervention strategies for weight loss and prevention of weight regain for adults. Med Sci Sports Exerc. 2001;33:2145–56. 11740312 10.1097/00005768-200112000-00026

[3] Bull FC, Al-Ansari SS, Biddle S, Borodulin K, Buman MP, Cardon G, . World Health Organization 2020 guidelines on physical activity and sedentary behaviour. Br J Sports Med. 2020;54:1451–62. 33239350 10.1136/bjsports-2020-102955 PMC7719906

[4] Gabriel BM, Zierath JR. The Limits of Exercise Physiology: From Performance to Health. Cell Metab. 2017;25:1000–11. 28467920 10.1016/j.cmet.2017.04.018

[5] MacInnis MJ, Skelly LE, Gibala MJ. CrossTalk proposal: Exercise training intensity is more important than volume to promote increases in human skeletal muscle mitochondrial content. J Physiol. 2019;597:4111–3. 31309577 10.1113/JP277633

[6] Soligon SD. Suspension training vs. traditional resistance training: effects on muscle mass, strength and functional performance in older adults. Eur J Appl Physiol. 2020. 10.1007/s00421-020-04446-x 32700098

[7] Zuo C, Bo S, Wang T, Zhang W. Functional and Traditional Resistance Training Are Equally Effective in Increasing Upper and Lower Limb Muscular Endurance and Performance Variables in Untrained Young Men. Front Physiol. 2022;13:868195. 36117686 10.3389/fphys.2022.868195 PMC9471151

[8] Little JP, Safdar A, Wilkin GP, Tarnopolsky MA, Gibala MJ. A practical model of low-volume high-intensity interval training induces mitochondrial biogenesis in human skeletal muscle: potential mechanisms. J Physiol. 2010;588:1011–22. 20100740 10.1113/jphysiol.2009.181743 PMC2849965

[9] Correia JC, Ferreira DMS, Ruas JL. Intercellular: local and systemic actions of skeletal muscle PGC-1s. Trends Endocrinol Metab. 2015;26:305–14. 25934582 10.1016/j.tem.2015.03.010

[10] Morash AJ, Vanderveken M, McClelland GB. Muscle metabolic remodeling in response to endurance exercise in salmonids. Front Physiol. 2014;5. 10.3389/fphys.2014.00452 PMC4240067 25484869

[11] Winder WW, Hardie DG. Inactivation of acetyl-CoA carboxylase and activation of AMP-activated protein kinase in muscle during exercise. Am J Physiol. 1996;270:E299–304. 8779952 10.1152/ajpendo.1996.270.2.E299

[12] Hardie DG. AMP-activated/SNF1 protein kinases: conserved guardians of cellular energy. Nat Rev Mol Cell Biol. 2007;8:774–85. 17712357 10.1038/nrm2249

[13] Hoffman NJ, Parker BL, Chaudhuri R, Fisher-Wellman KH, Kleinert M, Humphrey SJ, . Global Phosphoproteomic Analysis of Human Skeletal Muscle Reveals a Network of Exercise-Regulated Kinases and AMPK Substrates. Cell Metab. 2015;22:922–35. 26437602 10.1016/j.cmet.2015.09.001 PMC4635038

[14] Rabinovitch RC, Samborska B, Faubert B, Ma EH, Gravel SP, Andrzejewski S, . AMPK Maintains Cellular Metabolic Homeostasis through Regulation of Mitochondrial Reactive Oxygen Species. Cell Reports. 2017;21:1–9. 28978464 10.1016/j.celrep.2017.09.026

[15] Boström P, Wu J, Jedrychowski MP, Korde A, Ye L, Lo JC, . A PGC1-α-dependent myokine that drives brown-fat-like development of white fat and thermogenesis. Nature. 2012;481:463–8. 22237023 10.1038/nature10777 PMC3522098

[16] Wang S, Pan J. Irisin ameliorates depressive-like behaviors in rats by regulating energy metabolism. Biochem Biophys Res Commun. 2016;474:22–8. 27079240 10.1016/j.bbrc.2016.04.047

[17] Wu J, Boström P, Sparks LM, Ye L, Choi JH, Giang AH, . Beige Adipocytes Are a Distinct Type of Thermogenic Fat Cell in Mouse and Human. Cell. 2012;150:366–76. 22796012 10.1016/j.cell.2012.05.016 PMC3402601

[18] Erickson HP. Irisin and FNDC5 in retrospect: An exercise hormone or a transmembrane receptor? Adipocyte. 2013;2:289–93. 24052909 10.4161/adip.26082 PMC3774709

[19] Fry AC. The Role of Resistance Exercise Intensity on Muscle Fibre Adaptations. Sports Med. 2004;34:663–79. 15335243 10.2165/00007256-200434100-00004

[20] Tesch PA. Skeletal muscle adaptations consequent to long-term heavy resistance exercise. Med Sci Sports Exerc. 1988;20:S132–4. 3057312 10.1249/00005768-198810001-00008

[21] Porter C, Reidy PT, Bhattarai N, Sidossis LS, Rasmussen BB. Resistance Exercise Training Alters Mitochondrial Function in Human Skeletal Muscle. Med Sci Sports Exerc. 2015;47:1922–31. 25539479 10.1249/MSS.0000000000000605 PMC4478283

[22] Salvadego D, Domenis R, Lazzer S, Porcelli S, Rittweger J, Rizzo G, . Skeletal muscle oxidative function in vivo and ex vivo in athletes with marked hypertrophy from resistance training. J Appl Physiol. 2013;114:1527–35. 23519233 10.1152/japplphysiol.00883.2012

[23] Tsuchiya Y, Ando D, Takamatsu K, Goto K. Resistance exercise induces a greater irisin response than endurance exercise. Metabolism. 2015;64:1042–50. 26081427 10.1016/j.metabol.2015.05.010

[24] Liu Y, Guo C, Liu S, Zhang S, Mao Y, Fang L. Eight Weeks of High-Intensity Interval Static Strength Training Improves Skeletal Muscle Atrophy and Motor Function in Aged Rats via the PGC-1α/FNDC5/UCP1 Pathway. Clin Interv Aging. 2021;16:811–21. 34040358 10.2147/CIA.S308893 PMC8139720

[25] Safdar A, Little JP, Stokl AJ, Hettinga BP, Akhtar M, Tarnopolsky MA. Exercise Increases Mitochondrial PGC-1α Content and Promotes Nuclear-Mitochondrial Cross-talk to Coordinate Mitochondrial Biogenesis. J Biol Chem. 2011;286:10605–17. 21245132 10.1074/jbc.M110.211466 PMC3060512

[26] Huh JY, Dincer F, Mesfum E, Mantzoros CS. Irisin stimulates muscle growth-related genes and regulates adipocyte differentiation and metabolism in humans. Int J Obes. 2014;38:1538–44. 10.1038/ijo.2014.42 24614098

[27] Fatouros IG. Is irisin the new player in exercise-induced adaptations or not? A 2017 update. Clin Chem Lab Med. 2018;56:525–48. 29127759 10.1515/cclm-2017-0674

[28] Folland JP, Williams AG. The adaptations to strength training: morphological and neurological contributions to increased strength. Sports Med. 2007;37:145–68. 17241104 10.2165/00007256-200737020-00004

[29] Hass CJ, Feigenbaum MS, Franklin BA. Prescription of resistance training for healthy populations. Sports Med. 2001;31:953–64. 11735680 10.2165/00007256-200131140-00001

[30] Kraemer WJ, Fragala MS. Personalize It: Program Design in Resistance Training. ACSM’s Health Fit J. 2006;10:7–17.

[31] Neufer PD, Bamman MM, Muoio DM, Bouchard C, Cooper DM, Goodpaster BH, . Understanding the Cellular and Molecular Mechanisms of Physical Activity-Induced Health Benefits. Cell Metab. 2015;22:4–11. 26073496 10.1016/j.cmet.2015.05.011

[32] Zuo C, Bo S, Li Q, Zhang L. The Effect of Whole-Body Traditional and Functional Resistance Training on CAVI and Its Association With Muscular Fitness in Untrained Young Men. Front Physiol. 2022;13:888048. 35694401 10.3389/fphys.2022.888048 PMC9174581

[33] Miyatani M, Yang P, Thomas S, Craven BC, Oh P. Bioelectrical impedance and dual-energy X-Ray absorptiometry assessments of changes in body composition following exercise in patients with type 2 diabetes mellitus. J Obes. 2012;2012:1–9. 10.1155/2012/953060 PMC3457637 23029604

[34] Arazi H, Damirchi A, Asadi A. Age-related hormonal adaptations, muscle circumference and strength development with 8 weeks moderate intensity resistance training. Ann Endocrinol (Paris). 2013;74:30–5. 23337018 10.1016/j.ando.2012.11.004

[35] American College of Sports Medicine, Riebe D, Ehrman JK, Liguori G, Magal M, editors. ACSM’s guidelines for exercise testing and prescription. Tenth edition. Philadelphia: Wolters Kluwer; 2018.

[36] Arazi H, Asadi A, Roohi S. Enhancing muscular performance in women: compound versus complex, traditional resistance and plyometric training alone. J Musculoskelet Res. 2014;17:1450007.

[37] Cohen J. A power primer. Psychol Bull. 1992;112:155–9. 19565683 10.1037//0033-2909.112.1.155

[38] Fiatarone MA. High-Intensity Strength Training in Nonagenarians: Effects on Skeletal Muscle. JAMA. 1990;263:3029. 2342214

[39] Zuo C, Li Q, Zhang L, Bo S. Effects of 6-Week Traditional and Functional Resistance Training on Arterial Stiffness and Muscular Strength in Healthy Young Men. Front Physiol. 2022;13:859402. 35309075 10.3389/fphys.2022.859402 PMC8924443

[40] Lagally KM, Cordero J, Good J, Brown DD, McCaw ST. Physiologic and metabolic responses to a continuous functional resistance exercise workout. J Strength Cond Res. 2009;23:373–9. 19197213 10.1519/JSC.0b013e31818eb1c9

[41] Huh JY, Mougios V, Skraparlis A, Kabasakalis A, Mantzoros CS. Irisin in response to acute and chronic whole-body vibration exercise in humans. Metabolism. 2014;63:918–21. 24814685 10.1016/j.metabol.2014.04.001

[42] Kim H, Lee HJ, So B, Son JS, Yoon D, Song W. Effect of Aerobic Training and Resistance Training on Circulating Irisin Level and Their Association With Change of Body Composition in Overweight/Obese Adults: a Pilot Study. Physiol Res. 2016;271–9. 26447516 10.33549/physiolres.932997

[43] Maté-Muñoz JL, Monroy AJA, Jodra Jiménez P, Garnacho-Castaño MV. Effects of instability versus traditional resistance training on strength, power and velocity in untrained men. J Sports Sci Med. 2014;13:460–8. 25177170 PMC4126279

[44] Anderson K, Behm DG. Trunk muscle activity increases with unstable squat movements. Can J Appl Physiol. 2005;30:33–45. 15855681 10.1139/h05-103

[45] Asanuma H, Pavlides C. Neurobiological basis of motor learning in mammals. Neuroreport. 1997;8:R1–6. 9141042

[46] Schoenfeld BJ, Peterson MD, Ogborn D. Effects of Low- vs. High-Load Resistance Training on Muscle Strength and Hypertrophy in Well-Trained Men. J Strength Cond Res. 2015;29:2954–63. 25853914 10.1519/JSC.0000000000000958

[47] Herzig S, Shaw RJ. AMPK: guardian of metabolism and mitochondrial homeostasis. Nat Rev Mol Cell Biol. 2018;19:121–35. 28974774 10.1038/nrm.2017.95 PMC5780224

[48] Jäger S, Handschin C, St.-Pierre J, Spiegelman BM. AMP-activated protein kinase (AMPK) action in skeletal muscle via direct phosphorylation of PGC-1α. Proc Natl Acad Sci USA. 2007;104:12017–22. 17609368 10.1073/pnas.0705070104 PMC1924552

[49] Guadalupe-Grau A, Rodríguez-García L, Torres-Peralta R, Morales-Álamo D, Ponce-González JG, Pérez-Suarez I, . Greater basal skeletal muscle AMPKα phosphorylation in men than in women: Associations with anaerobic performance. Eur J Sport Sci. 2016;16:455–64. 26305090 10.1080/17461391.2015.1063701

[50] Ponce-González JG, Ara I, Larsen S, Guerra B, Calbet JAL, Helge JW. Effect of regional muscle location but not adiposity on mitochondrial biogenesis-regulating proteins. Eur J Appl Physiol. 2016;116:11–8. 26269447 10.1007/s00421-015-3232-7

[51] Zhang G, Zhu Y, Zeng F. Influence on Ratio of AMP/ATP and AMPK Activity in Rat’s Skeletal Muscle by Different Exercise Intensity. China Sport Science and Technology. 2008;44:19–23.

[52] Dreyer HC, Fujita S, Cadenas JG, Chinkes DL, Volpi E, Rasmussen BB. Resistance exercise increases AMPK activity and reduces 4E-BP1 phosphorylation and protein synthesis in human skeletal muscle: AMPK, muscle protein synthesis and resistance exercise. J Physiol. 2006;576:613–24. 16873412 10.1113/jphysiol.2006.113175 PMC1890364

[53] Rasmussen BB, Winder WW. Effect of exercise intensity on skeletal muscle malonyl-CoA and acetyl-CoA carboxylase. J Appl Physiol. 1997;83:1104–9. 9338417 10.1152/jappl.1997.83.4.1104

[54] Wojtaszewski JF, Nielsen P, Hansen BF, Richter EA, Kiens B. Isoform-specific and exercise intensity-dependent activation of 5′-AMP-activated protein kinase in human skeletal muscle. J Physiol. 2000;528(Pt 1):221–6. 11018120 10.1111/j.1469-7793.2000.t01-1-00221.x PMC2270117

[55] Jessen N, Pold R, Buhl ES, Jensen LS, Schmitz O, Lund S. Effects of AICAR and exercise on insulin-stimulated glucose uptake, signaling, and GLUT-4 content in rat muscles. J Appl Physiol (1985). 2003;94:1373–9. 12496137 10.1152/japplphysiol.00250.2002

[56] Hooshmand-Moghadam B, Eskandari M, Golestani F, Rezae S, Mahmoudi N, Gaeini AA. The effect of 12-week resistance exercise training on serum levels of cellular aging process parameters in elderly men. Exp Gerontol. 2020;141:111090. 32919015 10.1016/j.exger.2020.111090

[57] Tobina T, Yoshioka K, Hirata A, Mori S, Kiyonaga A, Tanaka H. Peroxisomal proliferator-activated receptor gamma co-activator-1 alpha gene expression increases above the lactate threshold in human skeletal muscle. J Sports Med Phys Fitness. 2011;51:683–8. 22212273

[58] Zhang Y, Uguccioni G, Ljubicic V, Irrcher I, Iqbal S, Singh K, . Multiple signaling pathways regulate contractile activity-mediated PGC-1 α gene expression and activity in skeletal muscle cells. Physiol Rep. 2014;2:e12008. 24843073 10.14814/phy2.12008 PMC4098736

[59] Aschenbach WG, Sakamoto K, Goodyear LJ. 5′ adenosine monophosphate-activated protein kinase, metabolism and exercise. Sports Med. 2004;34:91–103. 14965188 10.2165/00007256-200434020-00003

[60] Aydin S, Kuloglu T, Aydin S, Kalayci M, Yilmaz M, Cakmak T, . A comprehensive immunohistochemical examination of the distribution of the fat-burning protein irisin in biological tissues. Peptides. 2014;61:130–6. 25261800 10.1016/j.peptides.2014.09.014

[61] Pekkala S, Wiklund PK, Hulmi JJ, Ahtiainen JP, Horttanainen M, Pöllänen E, . Are skeletal muscle *FNDC5* gene expression and irisin release regulated by exercise and related to health?: FNDC5, irisin and exercise. J Physiol. 2013;591:5393–400. 24000180 10.1113/jphysiol.2013.263707 PMC3936375

[62] Lin W, Ju L, Wu J, Weng X. A new myokine-Irisin and exercise. J Guangzhou Sport Univ. 2016;36:87–90.

[63] García-Fontana B, Reyes-García R, Morales-Santana S, Ávila-Rubio V, Muñoz-Garach A, Rozas-Moreno P, . Relationship between myostatin and irisin in type 2 diabetes mellitus: a compensatory mechanism to an unfavourable metabolic state? Endocrine. 2016;52:54–62. 26438394 10.1007/s12020-015-0758-8

[64] Hee Park K, Zaichenko L, Brinkoetter M, Thakkar B, Sahin-Efe A, Joung KE, . Circulating Irisin in Relation to Insulin Resistance and the Metabolic Syndrome. J Clin Endocrinol Metab. 2013;98:4899–907. 24057291 10.1210/jc.2013-2373 PMC3849667

[65] Schiaffino S, Dyar KA, Ciciliot S, Blaauw B, Sandri M. Mechanisms regulating skeletal muscle growth and atrophy. FEBS J. 2013;280:4294–314. 23517348 10.1111/febs.12253

[66] Hayashi C, Toyoda H, Ogata S, Okano T, Mashino S. Long-term participation in community-based group resistance exercises delays the transition from robustness to frailty in older adults: a retrospective cohort study. Environ Health Prev Med. 2021;26:105. 34670491 10.1186/s12199-021-01028-x PMC8529757

